# Reflections of nurses and primary healthcare managers on integrating hospital at home into public primary healthcare services: a Norwegian focus group study

**DOI:** 10.1080/02813432.2024.2373310

**Published:** 2024-07-02

**Authors:** Lillian Karlsen, Bente Prytz Mjølstad, Bjarte Bye Løfaldli, Anne-Sofie Helvik

**Affiliations:** a The Centre for Health Innovation, Kristiansund, Norway; b Department of Medicine and Health Sciences, Department of Public Health and Nursing, Norwegian University of Science and Technology, Trondheim, Norway; c General Practice Research Unit, Department of Public Health and Nursing, Norwegian University of Science and Technology, Trondheim, Norway

**Keywords:** HaH, home care services, hospital-based, primary health care services, integrated care, rural nursing

## Abstract

**Background:**

Hospital at home (HaH) is an innovative approach to healthcare delivery that brings specialized services to patients’ homes. HaH services are typically available in urban areas where hospitals can easily reach nearby patients. An integrated care model that utilizes the public primary healthcare system may extend HaH services to include patients residing further away from hospitals. However, there is limited evidence of primary healthcare employees’ views on integrating HaH care into primary healthcare services. This study aimed to explore the reflections of primary healthcare employees on integrating HaH care into primary healthcare services.

**Methods:**

Ten focus group interviews were conducted with homecare nurses and managers of primary healthcare services in five municipalities in Mid-Norway. Reflexive thematic analysis was used to analyze the data.

**Results:**

The analysis resulted in three key themes regarding the integration of HaH care into primary healthcare. Participants discussed how they capture the distinctiveness of HaH care within the primary healthcare landscape. Moreover, they identified that the introduction of HaH care reveals opportunities to address challenges. Lastly, the study uncovered a strong primary healthcare commitment and a sense of professional pride among the participants. This resilience and dedication among primary healthcare employees appeared as an incentive to make the integration of HaH work.

**Conclusions:**

This study offers valuable insights into integrating HaH into primary healthcare services, highlighting opportunities to address challenges. The resilience and dedication of primary healthcare employees underscore their commitment to adapting to and thriving with HaH care. To establish a sustainable HaH care model, it is important to address geographical limitations, consider the strain on providers, maintain robust relationships, enhance funding, and formalize decision-making processes.

## Introduction

Healthcare services face significant challenges. The increasing demand for health care coupled with resource scarcity and tighter budgets necessitates a transformation to ensure sustainability [1]. A key strategy employed by several national health authorities involves redistributing roles and tasks from specialized healthcare services to primary healthcare to minimize hospital stays and establish viable alternatives to traditional inpatient care [2].

A hospital at home (HaH) represents an innovative care strategy supporting this development [3], enabling eligible patients to receive specialized treatment and care at home [4]. Previous studies have suggested that this model of care not only improves patient experiences [5] but also promotes population health [6] and reduces healthcare costs [7]. However, evaluating the effectiveness is challenged by the diversity of models, which vary in interventions, types of patients, staff composition, funding, and program components [8,9].

Until recently, HaH care was typically available to patients in urban areas [10], where hospital staff provided services outside the hospital [11,12]. This approach has geographical limitations as individuals living farther away from the hospital are often unable to access this high-quality model of care [12].

The Norwegian healthcare system, rooted in the Nordic welfare model [13], relies heavily on public funding (86%) and healthcare planning and delivery shared between the national and local governments [14]. Specialized healthcare is managed by four regional health authorities, whereas municipalities (357) oversee primary care and social services [15]. Although both levels of care are public, they are funded differently and governed by different laws [15,16]. Norway features well-performing healthcare services and maintains a high number of public healthcare personnel compared with other European countries [14]. Additionally, self-reported unmet medical needs were very low [14]. Still, fragmentation and coordination issues persist [16–18]. Moreover, Norway’s extensive geography and sparse population pose challenges related to the geographic distribution of healthcare resources [15,16,19].

This study examined an integrated HaH care model in Mid-Norway that aligns with Norwegian healthcare policies [16,19–21]. In this model, adult patients receive specialized treatment and care in their own homes, even if they reside further away from the hospital. Currently, treatment is limited to intravenous antibiotic therapy. Achieving this model requires collaboration between healthcare professionals from both specialized and primary healthcare services while emphasizing the involvement of patients and their family caregivers [22].

Integrated care services have been shown to alleviate care fragmentation [23], mitigate geographical disparities [24,25], and improve the quality of care [25]. However, contextual factors hindering the realization of these objectives within integrated care approaches have been identified [26]. As HaH is traditionally an initiative within specialized healthcare services, there is a lack of knowledge and understanding of the aspects surrounding an integrated care approach utilizing the primary healthcare system [12].

The transformation of healthcare services has led to the expansion of specialized tasks in primary healthcare [27,28]. However, this shift within the Norwegian healthcare system may not have been fully accompanied by the transfer of necessary resources and knowledge from hospitals to primary healthcare services [29–31]. This gap could potentially strain primary healthcare employees [30,32]. Norwegian municipalities with few inhabitants and long distances from hospitals are particularly vulnerable and face challenges due to limited resources, expertise, and recruitment [16,33]. A high workload is associated with staff burnout and may compromise the safety and quality of the care provided [34]. Although previous studies have reported high job satisfaction [35] and a low level of burnout [36] among HaH staff, these findings do not reflect the context in which public home care nurses constitute the major workforce in the care model.

To better understand the process of integrating HaH care into primary care, we aimed to gain insights into how key stakeholders involved in planning and providing primary healthcare services perceive this process. This investigation may identify areas that require improvement to establish a sustainable care model. To our knowledge, this is the first study solely focusing on the crucial reflections of homecare nurses and primary healthcare managers. This includes their expectations, opinions, and hands-on experience regarding the integration of HaH care for adults into public primary healthcare services.

This study aimed to explore the reflections of primary healthcare employees on integrating HaH care into primary healthcare services.

## Materials and methods

### Design

This qualitative study employed focus group interviews among homecare nurses and primary healthcare service managers. We aimed to uncover a broad range of insights and experiences that might otherwise remain hidden, emphasizing the vital role of group interactions in stimulating participants’ reflections [37]. We chose reflexive thematic analysis (TA) guided by the relativist view of reality as contingent, local, and multiple [38]. This perspective acknowledges that interpretations of data are context-dependent and influenced by dynamics within the group setting [39]. We adhered to the COREQ guidelines [40] for reporting this qualitative study (Additional file 1).

### Setting

The HaH model examined in this study represents an integrated care approach implemented in a region in Mid-Norway between 2018 and 2022, relying on collaboration between specialized and primary healthcare services to extend access to this care model. This inter-organizational collaborative effort aims to provide specialized home treatment for eligible adults with severe infections necessitating long-term intravenous antibiotic therapy (2–8 weeks).

Treatment was initiated during the hospital stay, followed by the training and empowerment of patients to continue therapy at home with support from local municipal homecare nurses. The use of electronic infusion pumps streamlines the treatment process, and the hospital pharmacy ensures medication preparation and delivery directly to the patients’ homes.

Throughout the treatment process, the attending hospital physician retains medical responsibility for the patient and develops a personalized treatment plan that directs both treatment and follow-up at home. Homecare nurses oversee treatment and follow-up activities tailored to a patient’s condition, need for assistance, and level of independence.

These nurses conducted scheduled home visits, which typically involved the administration of antibiotics, care for the central venous catheter, monitoring the patient’s condition, performing various measurements of patient health status, and communicating with hospital staff through electronic messages or telephone calls. Additionally, personnel at the municipal response center oversee patient safety alarms and coordinate immediate interventions in accordance with protocols. General practitioners in the primary healthcare system are not actively engaged in the HaH care model.

### Participant selection and sample

We employed purposeful sampling [41] and invited municipalities in Mid-Norway to participate in this study at various stages of implementing the HaH model. To ensure demographic diversity, LK and BBL identified five municipalities of diverse sizes, locations, and distances from hospitals (Table 1).

**Table 1.** Information about the participating municipalities (*n* = 5).

Municipality	Population	Location (urban or rural)	Stage of implementation
M1	<10,000	Rural	Middle
M2	>20,000	Urban	Middle
M3	>20,000	Urban	Early
M4	<10,000	Rural	Early
M5	<10,000	Rural	Early

LK contacted key personnel in the municipalities via email or telephone. These contact persons were asked to distribute invitations to healthcare managers and providers for participating in focus group interviews, aiming to achieve diversity in terms of age, sex, and work experience. All contacted municipalities, with the consent of their healthcare managers, agreed to participate, resulting in a total sample of 38 participants. Interviews were conducted between November 2021 and April 2022.

In each of the 5 municipalities, participants were divided into 2 focus groups: managers (comprising administrative managers overseeing various municipal healthcare services and operational managers responsible for home healthcare services) and clinicians (consisting of registered nurses (RN) in home healthcare services), resulting in a total of 10 focus groups (Table 2). This composition focused on homogeneity within each group to avoid hierarchical influences [37]. Additionally, we opted for intra-municipal grouping based on both practical considerations and the assumption that participants being familiar with each other would leverage a wider range of shared experiences compared with interactions with unknown persons [42].

**Table 2.** Information about the participants in the focus groups (*n* = 38).

Focus Group	Current work position	Sex	Average (range) age (years)	Average (range) years of work experience in current position
Managers (*n* = 19)	12 Administrative managers* 7 Operational managers**	17 women 2 men	54 (33–65)	8 (1–25)
Clinicians (*n* = 19)	19 Registered nurses (RN)	18 women 1 man	41 (23–58)	10 (1–30)

* *Administrative managers overseeing various healthcare services in the municipalities*.

** *Operational managers responsible for the home healthcare services in the municipalities*.

### Data collection

LK and ASH developed a semi-structured interview guide with open-ended questions, designed for this study, by integrating insights from our previous research and clinical experience (Additional file 2). The interview themes included the participants’ conceptual understanding of HaH, its organization, its impact on primary healthcare, and their expectations for its expansion. The guide underwent peer discussions but was not subjected to pilot testing.

All interviews were conducted at a public office in the municipality. LK moderated the interviews, and a research assistant documented the non-verbal communication and group dynamics. To ensure diverse perspectives, the moderator encouraged the participants to share various thoughts and reflections with all opinions valued [43]. Following each interview, LK and the assistant discussed the first impression of the group process and wrote reflective notes [38]. The interviews lasted between 49–89 min each and were audio recorded. The interviews were transcribed verbatim by LK and an external transcriber.

### Data analysis

We used reflexive thematic analysis adhering to the six analytical steps described by Clarke and Braun [38]. To enhance our analysis, we incorporated pertinent group interaction data that were deemed relevant to our findings by including quotes featuring comments from several participants [44]. This approach allowed us to observe interactions and gather information on how participants responded to each other’s statements, providing valuable information on the course of the group discussions [43].

In line with the first phase of familiarization, LK read all interview transcripts and reflective notes several times to gain an overall impression of the dataset, considering the group dynamics. ASH, BMP, and BBL reviewed the selected transcripts to familiarize themselves with the data. During the coding phase, LK codes the entire dataset using concise, data-driven, and mostly semantic phrases. These initial codes were clustered into candidate themes to identify and illustrate patterns in phase three. ASH, BMP, and BBL provided feedback on the coding matrix and initial themes. Throughout phase four, all researchers actively participated in the interpretation, development, and review of themes, engaging in shared reflections to understand the significance of the observed patterns and their broader implications. Following collective discussions, LK refined and named the three themes in phase five. This involved iteratively returning to the codes, transcripts, and reflective notes to ensure that these themes encapsulated crucial insights from the dataset and reflected the group contexts. Finally, LK drafted the initial report and identified illustrative quotations. The quotations were modified and condensed to exclude superfluous text and have been subsequently proofread by an experienced language editor. Refinement and further iterations were collaborative efforts involving contributions from all researchers.

Our analysis did not have a comparative intention, and we collectively analyzed the entire dataset. Nevertheless, we remained open to identifying patterns, including differences and similarities, between groups [45].

### Researcher backgrounds/research group

The first author, LK, is a nurse specializing in cancer care and a PhD candidate. She moderated all focus group interviews. Authors ASH, BMP, and BBL are senior researchers with backgrounds in nursing, general practice, and neuroscience, respectively. ASH and BMP have extensive experience with qualitative research methodologies. Both LK and BBL were familiar with the study setting, and LK had professional acquaintances with a few participants. Participants were informed of the interviewer’s professional background and the research group’s objectives.

## Results

In the analysis, we generated three themes: the capturing of the distinctiveness of HaH care within the primary healthcare landscape, the introduction of HaH care reveals opportunities to meet challenges, and primary healthcare commitment and professional pride, an incentive to make it work.

### Capturing the distinctiveness of HaH care within the primary healthcare landscape

During the focus group discussions, all participants attempted to position HaH within the primary healthcare landscape. Homecare nurses attempt to distinguish clinical tasks in HaH from their regular practice by asking questions about the novelty and uniqueness of the treatment and care inherent in this new care model. They recalled instances where similar care was delivered without being labeled HaH, leading to confusion.

"It feels like what we’ve been doing for years, just without labeling it as HaH. Patients don’t arrive here fully treated from the hospital; rather they come with ongoing treatment and then continue their care within the municipality. Essentially, it’s been happening in a similar way, even before this new term came into play." (Sarah, Nurse, Municipality 5)

All participants noted that the integration of HaH aligned with the ongoing transformation of primary healthcare services, reflecting a shift towards more advanced clinical practice in municipalities. Procedures such as the administration of intravenous antibiotics, management of central venous catheters, and operating infusion pumps were not novel for homecare nurses.

While acknowledging the similarities between their current practices and those associated with HaH care, participants also recognized the differences rooted in the system, organization, and inherent responsibilities of this new care model. The most prominent distinction was the assignment of medical responsibility to hospital physicians rather than to general practitioners in primary healthcare.

There was a discrepancy between the participants’ initial expectations of HaH care and their actual experiences with it. Initially, many participants across both groups associated it primarily with advanced palliative care, expecting its application in managing severely ill patients with complex medical conditions. However, participants who were most familiar with HaH care observed that their experiences contradicted these assumptions. The following conversations among homecare nurses underscored a clear consensus within the focus group concerning differences in the perceived duration and intensity of care provided to HaH patients compared to their typical patient groups:

Sophia: The patients in HaH are often not seriously ill. It’s more like a temporary setback. They are not necessarily ill in the usual sense.

Elisabeth: No, in HaH we have been in and carried out a specific task. But with our other patients, that is a bit of the difference, then there is often more involved. They need more help. With the HaH patients we have attended to, we completed our task and left. That was it.

Sophia: A clear start and a finish.

Elisabeth: Once the antibiotic treatment was finished, that was the end. Closed case.

Katherine: Terminated.” (Nurses, Municipality 3)

### Introducing HaH care reveals opportunities to meet challenges

Most participants in both groups expressed positive attitudes toward the HaH care model. They reported positive collaboration with hospital staff regarding these patients, viewing HaH as an opportunity to strengthen their overall relationship with specialized healthcare services. Being involved in early-stage planning for patient enrollment was embraced, providing them with the opportunity to decline such patients if their services lacked the necessary competency or if the workload was too high at the time. This represents a novel approach:

“We are used to facing situations where rapid discharge decisions are made, which compromises the quality of our service. Unlike institutional settings with limited bed capacities, our home care lacks clear limits, leaving us unable to decline care.” (Emma, Nurse, Municipality 2)

While participants grappled with ethical dilemmas when considering whether to prevent patients from receiving HaH care, they found empowerment in their ability to decline such patients if the quality of care was compromised.

In contrast, participants with extensive HaH care experience noted a growing demand to accept HaH care cases; sparking discussions on the appropriateness of primary healthcare services that should bear full responsibility for the follow-up of these patients. Those from municipalities near the hospital argued that the hospital staff should assist in providing follow-up care, particularly when primary healthcare services are unable to do so. In contrast, participants from rural municipalities were more accepting of these challenges, attributing them to the considerable distances to hospitals, which hindered hospital staff from providing such care.

The homecare nurses mostly experienced being well prepared by hospital staff before HaH patients returned home, benefiting from timely preparation, and being offered training if needed. This dialogue illustrates their appreciation for having sufficient time to prepare for the new patients:

Emma: That was the best thing with HaH. We received a thorough introduction and almost a week’s notice, which allowed us to plan effectively. Everything was well-organized.

Maya: That sounds truly wonderful.

Emma: Absolutely, it’s exactly like that, oh!

Laura: What’s happening here? Can it be like that?

Victoria: That’s not how our everyday life is…”(Nurses, Municipality 2)

Although homecare nurses were generally familiar with the tasks inherent in HaH, there were significant intervals between instances of their performance. Thus, having the opportunity to acquire or refresh their skills is important for them to feel confident when facing clinical situations alone in their homes. This confidence among homecare nurses fostered positive attitudes toward HaH.

Furthermore, most participants in both groups suggested that HaHs have the potential to contribute to an overall increase in competence within their municipalities. They perceived the expansion of specialized tasks as an opportunity for nurses to use their expertise more extensively, potentially aiding municipal recruitment efforts.

Despite recognizing opportunities, all participants stressed the need for appropriate resource allocation to primary healthcare services to support the sustainable expansion of this care model. Moreover, some managers emphasized the importance of adequate financial incentives as a prerequisite for continuing the follow-up of HaH patients:

Jan: We need to reach an agreement on the financial framework because it really affects us.

Anna: That’s a crucial point Jan. These patients belong to the hospital, generating DRG points (Diagnostic-related group points used for reimbursement) that boost its revenue. As a municipality, we should establish stricter criteria on this matter if we are to expand our involvement. Otherwise, hospitals thrive while we’re left with deficits.” (Managers, Municipality 1)

### Primary healthcare commitment and professional pride; an incentive to make it work

Many participants reported minimal resistance to carrying out the treatment and care tasks inherent in the HaH care model, demonstrating a positive willingness to adapt and embrace change.

During lively conversations, the homecare nurses discussed how to constantly embrace new tasks without hesitation:

Nina: We welcome these patients with open arms. I don’t think that any hospital department has the impression that our municipality doesn′t want our patient’s home, no.

Eva: No, we mostly take them home … yes. (laughs) almost all … not far off.

Nina: And that is, of course, if they call from the medical department and say: “We should have had a blood transfusion.” “Oh yeah, yeah, okay.”

Moderator: Okay, then can you do it?

Nina: Yes, it is like that … Then someone does it.” (Nurses, Municipality 1)

In addition to an overall strong municipal commitment among all participants, several underscored their shared responsibility in relieving pressure on hospitals by providing specialized treatment and care within the community. Moreover, several homecare nurses expressed personal fulfillment in providing home-based care to this new patient group: “*You see, it’s a pleasure to allow such patients to avoid being in the hospital. And to see how good it is for them to be at home.”* (Maria, Nurse, Municipality 4)

Both managers and homecare nurses, especially those in rural districts, outlined strategies developed within the municipality to address challenges related to assuming new tasks and roles inherent in HaH care despite limited resources. In one scenario, when they encountered a temporary lack of the necessary expertise in a patient’s home, they arranged for the transport of the patient to a nearby short-term nursing ward. A registered nurse was always available to perform the necessary procedures.

Homecare nurses shared a deep sense of empathy, stemming from their first-hand experiences with the inherent vulnerability of standing alone, providing specialized treatment, and providing care alone in patients’ homes. Drawing on these shared experiences, they established mutual alliances to offer collegial support to each other. This involved intensifying their presence and devising supportive strategies, even though this meant extending beyond their professional duties. As one nurse expressed: “*We push ourselves a little far on our free time… if things are to happen. Because we know that it’s never fun if things happen and you’re there all alone.”* (Sylvia, Nurse, Municipality 5)

## Discussions

This study situates HaH within the broader context of primary healthcare services. Participants’ reflections on integrating HaH care into the primary healthcare system ranged from initial expectations influenced by the ongoing development of primary healthcare services to post-utilization experiences of caring for such patients. They observed a blurred distinction between HaH clinical work and existing practices, viewing it as an organizational reform. Their experiences highlighted opportunities within this care model to address primary healthcare constraints. Furthermore, the study revealed strong municipal commitment and professional pride among primary healthcare employees, which served as incentives to adapt and find solutions.

This study revealed a disparity between what the participants expected, and the actual level and duration of care needed by these patients. Initially, HaH was associated with severely ill patients requiring complex care and advanced technologies. Notably, the participants’ experiences revealed that these patients demanded less intensive care than long-term patients currently managed by homecare nurses. This contrasts with the findings of Vaartio-Rajalin et al. [46] who suggested significant demands on HaH staff, including public home care nurses, highlighting the need for advanced clinical skills. This disparity may be attributed to the early phase of HaH implementation in our study, which was characterized by stringent inclusion criteria and careful patient selection by hospital clinicians [47,48]. In addition, variations in patient populations may contribute to this inconsistency [49]. Unlike Vaartio-Rajalin et al. [46], our study did not include patients requiring palliative care who typically have complex needs, resulting in a heavy workload [50]. Another factor to consider is the familiarity of Norwegian homecare nurses who perform advanced clinical assessments and procedures [51], reflecting the transfer of specialized care tasks from hospitals to primary healthcare services [20,32].

The study revealed predominantly positive attitudes toward HaH, suggesting that the integrated care model may hold promise for addressing the common challenges often faced by primary healthcare employees. Prior research has suggested that professionals in primary care often feel less valued in dialogue with specialized care services [52], and inter-organizational collaboration may have the potential to favor certain forms of clinical knowledge [53]. Interestingly, our findings suggest that collaboration among HaH may have empowered primary healthcare providers and encouraged a sense of local autonomy. This observation aligns with the findings of Karacaoglu and Leask [35], who reported that interprofessional HaH staff members felt valued and respected regardless of their position.

These findings suggest that the integrated HaH care model has the potential to enhance the overall expertise and attractiveness of primary healthcare services, which is particularly important given the growing challenges of recruiting nurses in municipalities [33]. HaH appears as an opportunity to transfers expertise from hospitals to primary healthcare services, addressing some of the gaps identified in studies that report a discrepancy between tasks and knowledge transfer [30,31]. However, it is essential to acknowledge this gap because caring for such patients adds to the workload of homecare nurses. Although patients with HaH may does not require high care intensity, homecare nurses must manage these tasks in addition to their regular responsibilities.

The lack of appropriate funding mechanisms may hinder the expansion of HaH services [48,54], particularly considering the complex nature of inter-organizational settings [55]. In Norway, specialized and primary healthcare services are funded by different systems [16,21], with primary healthcare currently not receiving reimbursement for HaH care tasks. Notably, our findings indicate a limited focus on funding concerns among the participants, as few focus groups have discussed this issue. This may be attributed to the limited representation of high-level administrative managers, as discussions were predominantly led by nurses and operational managers focusing on the practical aspects of HaH care. Achieving a sustainable integrated HaH care model seems to require a more equitable distribution of financial burdens and benefits among stakeholders across organizations [55].

Despite facing capacity challenges and varying experiences in previous collaborations with specialized healthcare services, our findings revealed a strong commitment among primary healthcare employees to adapt and effectively manage HaH care. Rooted in a municipal identity characterized by collective responsibility and professional pride, these professionals also derive personal fulfillment from providing care to residents in their homes instead of hospitalization. This perspective highlights the healthcare professionals’ ability to adjust, adapt, and maintain adequate work performance [56], as evidenced by primary healthcare workers, even in extreme situations, such as the COVID-19 pandemic [57,58]. Professional resilience, associated with traits, such as accepting uncertainty and finding personal meaning [59], aligns with our findings in the context of HaH care, particularly prevalent in rural areas characterized by heavy workloads and pressure [56,59,60].

Our findings support this aspect, as participants in rural municipalities appeared more open to integrating HaHs into primary healthcare services and finding solutions that extended beyond their professional duties. While this resilience appears to offer an opportunity to advance the integrated care model and expand HaH access for patients residing further away from hospitals, it is important to acknowledge its potential to mask the growing disparity between the demand for primary healthcare and the resources available in these services.

### Methodological considerations

The use of focus groups proved effective in gathering insights from a diverse range of primary health care employees engaged in the HaH care model, enabling a comprehensive understanding of various perspectives. However, the study did not explore the role of GPs as key stakeholders in the primary healthcare system. Future studies should consider these perspectives.

Choosing focus groups over individual interviews was advantageous because of the novelty of the HaH model and the limited number of experts in the field. The focus group composition fosters trustworthiness by reducing hierarchical barriers, creating a relaxed atmosphere, and encouraging active discussions among participants [37,61]. This allowed participants to explore their reflections and develop distinct viewpoints through interactions with their peers [62].

The imbalance in sex led us to reflect on whether the presence of more men in the focus groups influenced the topics discussed. Groups with male participants tended to raise financial concerns. Similar, including more administrative managers at the highest level may have influenced the topics brought up. Although we did not set out comparative intentions, we observed few differences between managers and nurses. This may be because most managers are closely involved in their field of practice. Additionally, group size also warrants consideration, as smaller groups facilitate active participant engagement, while larger groups may have provoked a broader range of responses [63].

The moderator had professional acquaintances with a few participants. While participants were informed about the interviewer′s background and research objectives prior to consenting to participate, these connections may have influenced some of the discussions. Nevertheless, the focus groups proceeded smoothly, and the moderator’s role was less active compared to that in an individual interview setting.

This study was conducted in a specific region of Norway, with participants chosen to represent municipalities that reflect demographic diversity and typical Norwegian characteristics. To improve the transferability of the study findings, we attempted to describe the context, participants, and settings thoroughly [64].

## Conclusions

This study offers valuable insights into the integration of HaHs into the landscape of primary healthcare services. Participants’ reflections illuminated opportunities to address common challenges. The resilience and dedication of primary healthcare employees underscore their shared commitment to adapt to and thrive in providing HaH care. While recognizing this resilience as an opportunity to advance the integrated HaH care model, particularly in rural municipalities, it is important to acknowledge its potential to mask the growing disparity between the demands placed on primary healthcare professionals and available resources. Moving forward, to establish a sustainable HaH care model integrated into the primary healthcare system and extend access, it’s important to consider the strain on primary healthcare employees. Efforts should include fostering robust collaborative relationships, enhancing funding mechanisms and reimbursement sustainability, and formalizing decision-making processes involving primary healthcare professionals.

## Supplementary Material

Supplemental Material
