# Supplementary material for: Reflections of nurses and primary healthcare managers on integrating hospital at home into public primary healthcare services: a Norwegian focus group study
Source: Scand J Prim Health Care. 2024 Jul 2;42(4):633–42. doi: 10.1080/02813432.2024.2373310 (PMC11552280; doi:10.1080/02813432.2024.2373310)
Supplement: Supplemental Material [file IPRI_A_2373310_SM1274.docx]

**Interview guide**

1. How familiar are you with Hospital at Home and the local care model in Mid-Norway?
2. What are your experiences with the integration of Hospital at Home into primary healthcare services?
3. Can you elaborate and reflect around the Hospital at Home care model?
4. Can you reflect on the clinical aspects of providing care to patients in Hospital at Home?
5. How do you perceive the impact of Hospital at Home?
6. Is there something you would like to add?
